# Preventable? Long-Term Care Policy Successes and Failures During COVID-19 Pandemic: A Scoping Literature Review

**DOI:** 10.1093/geroni/igab046.3081

**Published:** 2021-12-17

**Authors:** Chaoran Wu, Aleksandra Zecevic, Maxwell Smith, Shannon Sibbald

**Affiliations:** 1 University of Western Ontario, London, Ontario, Canada; 2 Western University, London, Ontario, Canada; 3 University of Western Ontario, University of Western Ontario, Ontario, Canada

## Abstract

The number of older adults who live in long-term care (LTC) is expected to increase worldwide. The COVID-19 pandemic has caused serious consequences in Canadian LTC homes, while homes in China and Japan reported minimal infection and death rates in residents. The differences in LTC policies may be one of the contributors. The purpose of this literature review was to identify elements of the LTC policies that might have impacted COVID-19 outcomes in LTC homes in Canada, China, and Japan. A scoping review was conducted following the framework proposed by Arksey and O’Malley. Scholarly articles and grey literature published between January 2015 and June 2020 were identified in six databases, four in English (CINAHL, Scopus, ProQuest, and PubMed), one in Chinese (CNKI), and one in Japanese (CiNii), using MeSH terms for LTC and health policy. Grey literature was identified using Google. Data were extracted, summarized and common themes identified through content analysis. A total of 52 articles and 26 grey sources were included in the review based on determined inclusion criteria. They were research articles, reviews, government or association reports, policy briefs, policy documents, and guides. Four common themes of challenges emerged: caregiver workforce, service provision, funding, and physical environments. Three sub-themes were identified for caregiver workforce and service provision. Differences in COVID-19 consequences in LTC homes in the three countries seem to be related mainly to the challenges with the caregiver workforce and the lack of funding. The result suggests Improvements of LTC policies are required, especially in Canada.

